# Advancing perspectives on the off-label use of anticancer drugs: an updated classification and exploration of categories

**DOI:** 10.3389/fphar.2024.1374549

**Published:** 2024-06-04

**Authors:** Xiaoyi Chen, Shunlong Ou, Jing Luo, Zhisan He, Qian Jiang

**Affiliations:** ^1^ School of Medicine, University of Electronic Science and Technology of China, Chengdu, Sichuan, China; ^2^ Department of Pharmacy, Sichuan Clinical Research Center for Cancer, Sichuan Cancer Hospital and Institute, Sichuan Cancer Center, Affiliated Cancer Hospital of University of Electronic Science and Technology of China, Chengdu, Sichuan, China; ^3^ Department of Pharmacy, The Second People’s Hospital of Yibin, Yibin, Sichuan, China

**Keywords:** off-label use, antineoplastic agents, definitions, categories, classification, update

## Abstract

To date, the definition that the off-label usage of drugs refers to the unapproved use of approved drugs, which covers unapproved indications, patient populations, doses, and/or routes of administration, has been in existence for many years. Currently, there is a limited frequency and prevalence of research on the off-label use of antineoplastic drugs, mainly due to incomplete definition and classification issues. It is time to embrace new categories for the off-label usage of anticancer drugs. This review provided an insight into an updated overview of the concept and categories of the off-label use of anticancer drugs, along with illustrating specific examples to establish the next studies about the extent of the off-label usage of anticancer drugs in the oncology setting. The scope of the off-label use of current anticancer drugs beyond the previous definitions not only includes off-label uses in terms of indications, patient populations, doses, and/or routes of administration but also off-label use in terms of medication course, combination, sequence of medication, clinical purpose, contraindications scenarios, etc. In addition, the definition of the off-label usage of anticancer drugs should be added to the condition at a given time, and it varies from approval authorities. We presented a new and relatively comprehensive classification, providing extensive analysis and illustrative examples of the off-label usage of antineoplastic drugs for the first time. Such a classification has the potential to promote practical adoption and enhance management strategies for the off-label use of antitumor drugs.

## 1 Introduction

Physicians must deal with both unmet clinical needs and fast-paced and abundant therapeutic advancement. Therefore, off-label uses are widespread across medical disciplines in real-world settings, especially in oncology, rare diseases, pediatrics, psychiatry (Stone et al., 2003; Pandolfini and Bonati, 2005; Hampton, 2007), etc., which existed for many years but are still a far-reaching problem in healthcare systems worldwide year on year. Although previous studies have addressed this significant dilemma of anticancer drugs (Poole and Dooley, 2004; Vickers et al., 2006), only a few surveys have been carried out to ascertain its true extent considering the complexity of off-label encounters. For example, one study conducted in Australia has shown that 85% of all cancer patients in the study were prescribed at least one drug that had not been fully tested by the regulatory approval process (Mellor et al., 2009), while the prevalence of the off-label use of cancer treatments among women with breast cancer in the United States was 55.4%, respectively (Hamel et al., 2015), varying according to the country and jurisdiction.

To attack the essence, each well-defined off-label use manner in oncology settings is the kernel and the key. To date, off-label use in drug labeling refers to the utilization of approved drugs for unapproved indications, patient populations, doses, and/or routes of administration without official approval. This definition has been established for many years (ASHP statement on the use of medications for unlabeled uses, 1992). However, there is currently a lack of extensive research on the prevalence and frequency of the off-label use of antitumor drugs, primarily due to incomplete definition and classification. Some studies are underway to explore the classification of such medications. For example, Levêque D primarily discussed the off-label use of anticancer drugs, covering various aspects such as the type or subtype of cancer, type of association, treatment lines, and duration of treatment (Levêque, 2008). While extracting data for a systematic review of the unapproved use of antineoplastic medications, it was divided into four categories: i) unapproved drug for a specific tumor group; ii) unapproved drug for a specific stage of disease (neoadjuvant, adjuvant, palliative, and curative); iii) unapproved line of treatment; and iv) modified application of the drug (e.g., dose, frequency, combination, and route of administration) (Saiyed et al., 2017).

Nevertheless, categories of the off-label usage of anticancer drugs that existed according to labeling information can be precise and far more than the relative definition mentioned above. In this review, we expanded and updated more refined classification categories for the off-label use of antitumor drugs by citing specific examples, presenting it for consideration by the scientific community. We also discussed the current limitations in categorizing the off-label use of antitumor drugs and proposed future directions for consideration.

The updated classification and exploration of the off-label uses of anticancer drugs would provide a valuable resource for researchers and clinicians, facilitating the identification of novel therapeutic options for cancer patients and driving improvements in the management of the off-label use of anticancer drugs by medical institutions and improved relevant laws and regulations.

## 2 Off-label usage of drugs is time-limited/varying

**FIGURE 1:**
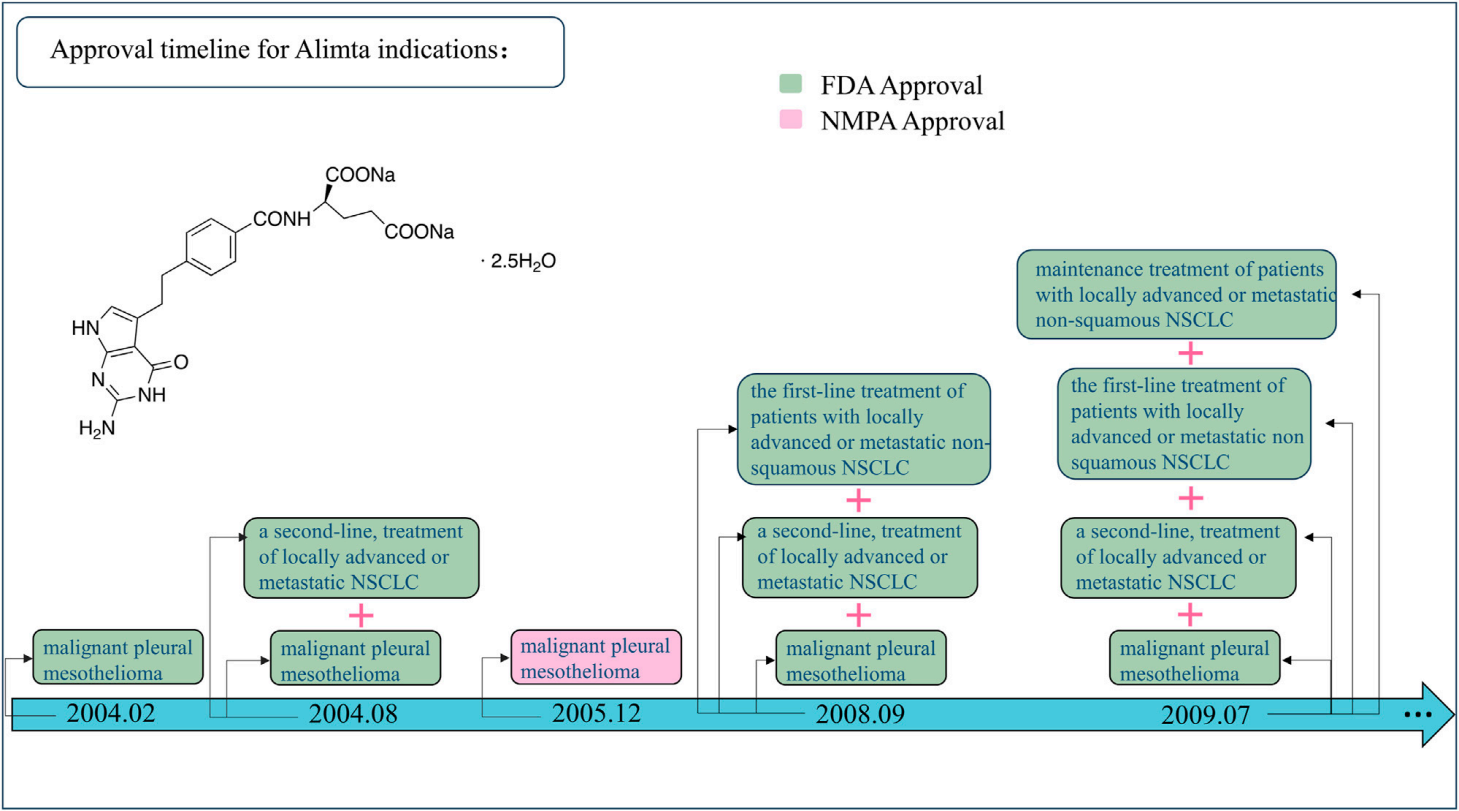
Approval timeline for Alimta indications. FDA, United States Food and Drug Administration; NMDA, National Medical Products Administration; NSCLC, non-small-cell lung cancer.

Off-label uses become advantageous in the oncology setting when high-quality scientific evidence is generated. Further research of compelling evidence is developed, promoting additional opportunities to incorporate it into labeling or restricting it to a narrow range. For example, in 2004, pemetrexed (Alimta^®^) was approved initially for the treatment of malignant pleural mesothelioma (Hazarika et al., 2004) and as the second-line treatment of locally advanced or metastatic non-small-cell lung cancer (NSCLC) (Rollins and Lindley, 2005). Then, the indication was expanded to the first-line treatment of the same type of NSCLC in 2008 (Ricciardi et al., 2009) and the maintenance treatment of the same in 2009 (Cohen et al., 2010) (Figure 1). Hence, the concept of off-label uses is time-limited and/or time-varying (Levêque, 2008); this is to say that it is first an ongoing process, which should be redefined over the course of time since substantial evidence is available. This situation is more common in new anticancer drugs, mainly targeted therapies, associated with different tumor types carrying the same mutation (Levêque, 2016), and immunotherapies, inducing T-cell response (Li et al., 2018), which is susceptible to be used in any kind of cancer.

## 3 Categories for the off-label usage of anticancer drugs should be reclassified

Indeed, the traditional definition (ASHP statement on the use of medications for unlabeled uses, 1992) of the off-label usage of anticancer drugs is the tip of the iceberg in practice; in many cases, newer categories should be addressed (Table 1).

**TABLE 1:** Reclassification of categorization for the unapproved utilization of anticancer drugs.

No.	Category	Content	Specific item	Example
1	Unapproved indications^a^	Different cancer types	Increases the type	Oxaliplatin is approved for colorectal cancer but used for non-Hodgkin’s lymphoma, esophageal cancer, biliary tract cancer, stomach cancer, ovarian cancer, pancreatic cancer, NSCLC, testicular cancer, transitional cell carcinoma of the bladder, and hepatocellular carcinoma in clinical practice (Motzer et al., 2012; Eroglu and Fruehauf, 2013; Gore et al., 2019; Benson et al., 2021; Ajani et al., 2022, 2023; Horwitz et al., 2022; Huang et al., 2022; Ren et al., 2022; Li et al., 2023)
		Extended indications with the same cancer type	Stage	Oxaliplatin combined with 5-Fu/leucovorin as an adjuvant therapy for adults with stage Ⅱ colon cancer (Baxter et al., 2022)
			Subtype	Trastuzumab could be used in HER2-negative instead of HER2-positive breast cancer from the NSABP B31 and the NCCTG N9831 trials (Ignatiadis et al., 2018)
			Line	Pyrotinib varied from the second-line drug for the treatment of advanced HER-2 positive breast cancer to the first-line treatment (Xu et al., 2022). Regorafenib approved for second-line therapy but used as a first-line agent alone for advanced hepatocellular carcinoma (Bai et al., 2023)
2	Unapproved dosage^a^	Single dose	Higher than approval dosage	High-dose carboplatin is used in patients with BRCA1 or BRCA2-associated metastatic breast cancer (Rottenberg et al., 2021)
			Lower than approval dosage	Pembrolizumab was given at a dose of 100 mg instead of the fixed dose of 200 mg in combination with chemotherapy (Chatterjee et al., 2016; Wang et al., 2023a)
			Different levels of expression of dosing	Fixed dose of trastuzumab prescribed instead of that adjusted for body weight (Levêque, 2008; Pivot et al., 2017)
		Frequency	Lower than approval frequency	Capecitabine given on a once-daily basis as opposed to a twice-daily regimen (Bachelot et al., 2013)
3	Unauthorized route of administration^a^	Modify routes	Increases routes	Alemtuzumab was given subcutaneously instead of intravenously (Lundin et al., 2002); intraperitoneal administration of cisplatin (Zhao et al., 2022); and subcutaneous injection of trastuzumab instead of intravenous infusion (Pivot et al., 2017; Heo and Syed, 2019; Denys et al., 2020)
			Change routes	Recombinant human endostatin is administered via infusion/pumps or intrathoracic perfusion instead of intravenous drip (Wang et al., 2020)
4	Off-label use in different patient groups^a^	Different pathophysiological conditions	Children and adolescents	Use of adult-approved drugs in children (Park et al., 2011, 2019; Granger et al., 2021)
			Physiological conditions in women	Toremifene for postmenopausal estrogen receptor-positive breast cancer patients can be extended to premenopausal breast cancer patients (Gradishar et al., 2022)
			Hepatic dysfunction	Use of vincristine was avoided instead of reducing the dosage by 50% when the serum bilirubin level exceeded 3 mg/dL (Superfin et al., 2007)
			Renal dysfunction	Sorafenib was adjusted in patients with mild, moderate, or severe kidney injury who did not undergo dialysis instead of not being adjusted (Miller et al., 2009)
5	Unapproved medication course	Dosing interval	Extends time	Defer treatment during the COVID-19 pandemic (de Azambuja et al., 2020; Dietz et al., 2020)
			Shortens time	Every 2 weeks instead of every 3 weeks for taxane drugs (Early Breast Cancer Trialists’ Collaborative Group EBCTCG (2019)
		Change days in a course of treatment	Extends days	Cisplatin from 75 mg/m^3^/d changed to 25 mg/m^3^/d for 3 consecutive days (Zhang et al., 2020)
		Increase the course of treatment	Increases the course of treatment	Bevacizumab combination chemotherapy regimen can be extended from 6 to 10 cycles (Aghajanian et al., 2015)
		Shorten the course of treatment	Shorten the course of treatment	Trastuzumab adjuvant therapy was reduced to 9 weeks (Conte et al., 2018)
6	Unlicensed combination	Drug approved as monotherapy but changed to a combination	Combined with other drugs	Raltitrexed combined with irinotecan for the treatment of metastatic colorectal cancer (Cheng et al., 2022)
		Drug approved in combination but given as a single agent	Single-drug administration	Bevacizumab monotherapy for the treatment of metastatic colorectal cancer (Hegewisch-Becker et al., 2015)
		Type of association	Changing the combination drug	Trastuzumab is given with vinorelbine instead of paclitaxel or docetaxel in untreated metastatic breast cancer (Andersson et al., 2011)
7	Unsanctioned sequence of medication	Infusion order	Changes the sequential order	Ifosfamide and cisplatin were administered simultaneously (Vanhoefer et al., 2000)
		Drug priority change	Does not require prior treatment	Nivolumab is used to treat metastatic NSCLC without prior treatment (Hellmann et al., 2019)
8	Unapproved clinical purpose	Broaden new clinical use	Addition for diagnostic use	Carboplatin skin test for diagnosing carboplatin allergies (Sliesoraitis and Chikhale, 2005)
			Addition for preventive use	Dexrazoxane is used to prevent heart toxicity caused by anthracycline beyond its recommendation for patients who have just started using anthracycline (Saleh et al., 2021)
9	Ultra-contraindications	Beyond limits	Ultra-contraindicated patient groups	Doxorubicin and cyclophosphamide used in mid/late-stage breast cancer (Germann et al., 2004; Amant et al., 2012; Loibl et al., 2012; Peccatori et al., 2013; Murthy et al., 2014)

^a^ The ASHP’s definition of off-label use.

### 3.1 Unapproved indications

Prescribing an approved anticancer drug beyond its licensed indications should be subdivided into two sectors: i) expanding to different cancer types, for example, oxaliplatin is approved for colorectal cancer but used for non-Hodgkin’s lymphoma, esophageal cancer, biliary tract cancer, stomach cancer, ovarian cancer, pancreatic cancer, NSCLC, testicular cancer, transitional cell carcinoma of the bladder, and hepatocellular carcinoma in clinical practice (Motzer et al., 2012; Eroglu and Fruehauf, 2013; Gore et al., 2019; Benson et al., 2021; Ajani et al., 2022, 2023; Horwitz et al., 2022; Huang et al., 2022; Ren et al., 2022; Li et al., 2023) and ii) expanding to a different stage (oxaliplatin with 5-Fu/calcium folinate is used as adjuvant therapy for stage II colon cancer in adults) (Baxter et al., 2022), subtype (trastuzumab for HER2-negative rather than HER2-positive breast cancer) (Ignatiadis et al., 2018), or treatment line of the same cancer type (pyrotinib switched from second-line to the first-line treatment for advanced HER2-positive metastatic breast cancer) (Xu et al., 2022). Regorafenib is approved for second-line therapy but used as a first-line agent alone for advanced hepatocellular carcinoma (Bai et al., 2023).

### 3.2 Unapproved dosage

The use of anticancer drugs beyond their approved dosage could be illustrated by two sectors: i) unapproved single dose (higher, lower, or different expression of dosing). For instance, compared with the approved dose, high-dose carboplatin-based chemotherapy has demonstrated curative potential in several patients diagnosed with BRCA1 or BRCA2-associated metastatic breast cancer (Rottenberg et al., 2021). Pembrolizumab is administered at a dose of 100 mg (2 mg/kg) instead of the fixed dose of 200 mg in combination chemotherapy (the KEYNOTE-001 study illustrated that the dose range of pembrolizumab is 0.005 mg/kg∼10 mg/kg, with maximum antitumor activity achieved at 2 mg/kg, and pembrolizumab administered through weight-based dosing and fixed-dose regimens had comparable pharmacokinetics [PK]) (Chatterjee et al., 2016). Pembrolizumab could potentially decrease the potential financial toxicity if given with PK guidance in patients with advanced NSCLC (Wang et al., 2023). As for the different levels of expression of dosing, a fixed dose of trastuzumab was prescribed instead of the dose adjusted for body weight in practice (Levêque, 2008; Pivot et al., 2017); ii) unlicensed frequency (lower), where the regimen consists of capecitabine (2,000 mg/m^2^ once daily on days 1–14 of each 21-day cycle) in combination with lapatinib (1,250 mg/d). A single-group phase 2 trial including 45 patients with previously untreated brain metastases from HER2-positive metastatic breast cancer has provided evidence supporting the use of capecitabine once a day as opposed to twice a day (Bachelot et al., 2013).

### 3.3 Unauthorized route for medication administration

The unauthorized route for medication administration has two ways in practice: i) broadening the routes, for example, subcutaneous injection of alemtuzumab instead of intravenous infusion for the treatment of refractory chronic lymphocytic leukemia (CLL) (Lundin et al., 2002), intraperitoneal infusion of cisplatin for the treatment of malignant pleural effusion caused by conditions like NSCLC (Zhao et al., 2022), or subcutaneous injection of trastuzumab instead of intravenous infusion for HER2-positive breast cancer (Pivot et al., 2017; Heo and Syed, 2019; Denys et al., 2020), and ii) substituting the approved routes, such as the administration of recombinant human endostatin injection through infusion/pouring into the pleural cavity instead of intravenous drip for the treatment of advanced NSCLC or malignant pleural effusion (Wang et al., 2020).

### 3.4 Off-label use in different patient groups

The utilization of approved anticancer medications beyond their licensed patient groups can be classified into different pathophysiological conditions. One of the scenarios is using adult-approved agents in pediatric patients: children in the United States with high-risk neuroblastoma undergo five intensive chemotherapy cycles using adult-approved drugs, including vincristine, cyclophosphamide, topotecan, doxorubicin, cisplatin, and etoposide (Park et al., 2011, 2019; Granger et al., 2021). Another scenario has occurred in different physiological conditions in women: toremifene is indicated for the treatment of estrogen receptor-positive/or unknown metastatic breast cancer in postmenopausal women but can be extended to premenopausal patients with breast cancer (Gradishar et al., 2022). The scenario that has occurred in patients with hepatic or renal dysfunction is supposed to be considered. For instance, the prescribing instructions for vincristine recommend reducing the dosage by 50% when the patient’s serum bilirubin level is above 3 mg/dL. However, some clinical practitioners advise against using this medication when the serum bilirubin level is above 3 mg/dL or the aminotransferase level exceeds three times the upper limit of the normal level (Superfin et al., 2007). Sorafenib is not recommended to be dose-adjusted for patients with mild, moderate, or severe renal impairment without undergoing dialysis. However, a phase I study suggested that for patients with a creatinine clearance (CrCl) of 20–30 mL/min, the initial dose of sorafenib should be reduced to 200 mg twice daily, whereas for patients undergoing dialysis, it should be reduced to 200 mg once daily (Miller et al., 2009).

### 3.5 Unapproved medication course

The use of an approved antineoplastic agent beyond its licensed regimen could be subdivided into four sectors: i) lengthen/shortening the treatment interval: the former situation where patients with cancer are frequently immunocompromised during the COVID-19 pandemic and acquiring COVID-19 may significantly affect the diagnosis and treatment of their primary disease. Breast cancer patients who are co-infected with COVID-19 may wish to consider putting off chemotherapy as an option to ensure the stability of their condition (de Azambuja et al., 2020; Dietz et al., 2020) and the latter situation, where taxane drugs are used every 2 weeks instead of every 3 weeks for adjuvant chemotherapy in breast cancer (Early Breast Cancer Trialists’ Collaborative Group [EBCTCG], 2019); ii) extending the number of days of medication within a course of treatment: for instance, replacing the usual dose of 75 mg/m^2^ administered on the first day of a 3-week cycle with a split dose over 3 days (25 mg/m^2^ each day on days 1–3) to treat advanced breast cancer with cisplatin may reduce the frequency of unwanted side effects such as nausea, vomiting, kidney toxicity, and hypomagnesemia (Zhang et al., 2020); iii) increasing the course of the treatment: the bevacizumab combination chemotherapy regimen can be extended from 6 to 10 cycles in patients with recurrent ovarian cancer that is sensitive to platinum (Aghajanian et al., 2015); and iv) shortening the course of the treatment: a comparison between 9 weeks and 1 year of trastuzumab adjuvant therapy in HER2-positive early breast cancer patients did not show non-inferiority, and the standard treatment also remains the 1-year trastuzumab regimen. Nevertheless, the 9-week regimen could lower the risk of severe cardiac toxicity. Therefore, patients with low recurrence risk or those experiencing cardiac events during the treatment could opt for the 9-week option (Conte et al., 2018).

### 3.6 Unlicensed combination

Combination therapy is a common form of antitumor regimen. Examples of such unlicensed medication use include i) changing monotherapy into a combination. Larotrectinib has been approved as a monotherapy for patients with advanced colorectal cancer who are unsuitable for 5-Fu/leucovorin calcium. However, the off-label use here is based on a phase II clinical trial that confirmed the efficacy, convenience, and tolerability of the larotrectinib and irinotecan combination as a chemotherapy regimen for the second-line treatment of metastatic colorectal cancer (Cheng et al., 2022); ii) changing the combination into a single agent. Bevacizumab is approved for the treatment of metastatic colorectal cancer when used in combination with fluorouracil-based chemotherapy. However, studies indicate the bevacizumab alone is non-inferior to standard fluoropyrimidine plus bevacizumab. Switching from combination therapy to monotherapy is still within the clinically acceptable range (Hegewisch-Becker et al., 2015); and iii) transformed type of association. Trastuzumab is approved to be used in association with paclitaxel or docetaxel for patients diagnosed with HER2-positive chemotherapy-naïve metastatic breast cancer, but research has shown that when used with vinorelbine for the treatment of untreated metastatic breast cancer, trastuzumab has significantly fewer adverse effects and can serve as a first-line alternative treatment (Andersson et al., 2011).

### 3.7 Unsanctioned sequence of medication

The sequential order of antineoplastic drugs can also involve unsanctioned use. One is altering sequential drug delivery, and another is no prior treatment in clinical drug priorities. The former example, such as the sequential use of cisplatin followed by ifosfamide, may exacerbate the myelosuppression, neurotoxicity, and nephrotoxicity of ifosfamide. However, research has shown that the concurrent administration of ifosfamide and cisplatin in chemotherapy for ovarian cancer patients can synergistically enhance efficacy (Vanhoefer et al., 2000). In addition, with the latter, for example, there is no prior therapy required for nivolumab. However, a study has shown that nivolumab plus ipilimumab is associated with better survival than chemotherapy-advanced NSCLC patients with varying levels of PD-L1 expression who have not received chemotherapy before (Hellmann et al., 2019).

### 3.8 Unapproved clinical purpose

The use of approved anticancer agents beyond their sanctioned clinical purpose could be divided into two sectors: i) additional diagnostic use. For example, carboplatin is mainly used in chemotherapy for ovarian cancer, small-cell lung cancer, non-small-cell lung cancer, head and neck squamous cell cancer, esophageal cancer, seminoma, bladder cancer, and mesothelioma, but it can also be used in skin tests to diagnose whether patients are allergic to carboplatin. The incidence of anaphylactic reactions was 27% for more than seven courses of carboplatin. For more than eight courses of treatment, the cumulative incidence of anaphylactic reactions was 44%. The highest number of carboplatin-induced anaphylactic reactions occurred during the 8th or 9th treatment cycle (Markman et al., 2003). A longer interval between chemotherapy cycles increases the risk of developing carboplatin allergic reactions (Tham et al., 2015). Therefore, it is advised to perform a skin test before the 6th cycle of carboplatin chemotherapy to ascertain the potential risk of a carboplatin allergy (Sliesoraitis and Chikhale, 2005), and ii) added preventive use. Dexrazoxane is used to prevent heart toxicity caused by anthracycline (Saleh et al., 2021), while the leaflet states it is not recommended for patients who have just started using it.

### 3.9 Ultra contraindications

It is generally considered unreasonable to use medications in contraindicated conditions as it may pose risks to a patient’s health. However, there are exceptions, such as the use of doxorubicin and cyclophosphamide in pregnant women, which are typically prohibited. Yet, anthracycline-based chemotherapy regimens have been utilized in pregnant patients with mid/late-stage breast cancer [chemotherapeutic regimens such as fluorouracil, doxorubicin, and cyclophosphamide (FAC regimen)] (Germann et al., 2004; Amant et al., 2012; Loibl et al., 2012; Peccatori et al., 2013). The decision to use medications in contraindicated conditions was based on a prospective single-group study that included 87 patients receiving fluorouracil, doxorubicin, and cyclophosphamide combination chemotherapy during mid/late-stage pregnancy, without any occurrences of stillbirth, spontaneous abortion, or perinatal death. Moreover, the majority of these children did not experience any severe neonatal complications (Murthy et al., 2014).

## 4 Discussion

To the best of our knowledge, the reasons behind the off-label use of drugs are multifaceted (10), stemming from the relatively narrow indications often specified on the approved label, a lack of approved drugs available for a certain disease or setting, and the desire to provide a promising new drug to a patient who might not have access through approval or a clinical trial. When the drug patent expires, pharmaceutical companies will no longer have any incentive to pursue label extensions (Casali and Executive Committee of ESMO, 2007).

However, the suitability of anticancer medications for off-label use remains controversial due to uncertainty around the clinical benefits and potential toxicities (Mullins et al., 2010; Irwin et al., 2012; Eguale et al., 2016), limited evidence to support clinical decision-making (Luo et al., 2023; Si and Ma, 2023), increased out-of-pocket costs for patients (Levêque, 2008; Mehr, 2012), and ethical concerns regarding the lack of informed consent (Zheng et al., 2017). So, in the oncology setting, off-label uses raise increased awareness. As we can see, implementing an ethically sound, logistically efficient, and financially prudent decision-making process to determine which off-label use of antitumor drugs should be covered by Medicare (Sox, 2009) still merits discussion on a categorical basis today.

Exploring the categories of the off-label use of anticancer drugs also has some challenges and future directions.

First, we need to pay attention to the fact that the premise of off-label drug use is reasonable. Previous studies on the frequency and prevalence of antitumor off-label drug use did not distinguish between them (Wang et al., 2013). Nevertheless, our research on off-label drug use is based on evidence and a reasonable premise. The unapproved label use does not necessarily mean a lack of evidence demonstrating the efficacy and safety of the used agent, and the supporting evidence for different unapproved indications may vary considerably both in extent and quality (Pfister, 2012). It could be distinguished between being supported by scientific evidence and having little evidence. Second, the definition of the off-label usage of anticancer drugs should be added to a condition of a given time, and it varies from approval authorities. (Saiyed et al., 2017). Off-label uses can be switched to on-label uses since compelling high-quality scientific evidence has become available over time, and the latter can also be switched to the former (Levêque, 2008). Third, to establish reliable data on the extent of such usage, exploring newer categories could, along with the state-of-the-art categories, lead to standardized administration in physician decision-making, law, hospital pharmacy management, reimbursement, and other directions related to the unapproved use of antineoplastic drugs, which may guide future investigations.

Additionally, it is not possible to fully explore all new categories of antineoplastic drugs for off-label use because the clinical requirements are intricate and varied, such as the requirements of patients with advanced cancer, multiple diseases, adherence, affordability, and tolerability, physician decision-making, and the same drug from different manufacturers or different specifications and dosage forms from the same manufacturer, which can also lead to the unapproved use of antineoplastic drug. Nevertheless, to promote the standardized management and rational use of unapproved anticancer drugs, the scope of categories on unapproved antineoplastic drug use can be further extended from electronic medical records, patient-reported outcome studies (Khan and Butler, 2022), and the real-world database from China (Wang et al., 2023).

A statement regarding the generalizability and limitations of this study are as follows.

While this study provides valuable insights into the off-label use of anticancer drugs, it is important to acknowledge certain limitations that may affect the generalizability of our findings. First, the data primarily relied on literature reviews and RCTs, which may introduce inherent biases and limitations associated with the included studies. Additionally, the scope of our study focused on a specific subset of cancers and may not fully represent the broader landscape of off-label drug use in oncology. Furthermore, variations in clinical practice and regulatory frameworks across different regions may impact the applicability of our findings to diverse healthcare settings.

Despite these limitations, our study offers a relatively comprehensive overview of the off-label use of anticancer drugs, highlighting the need for further research and regulatory guidance in this evolving field. Future studies should address these limitations by incorporating larger sample sizes, diverse patient populations, and real-world data to enhance the generalizability of findings and effectively inform clinical practice.

## 5 Conclusion

New categories are important for the researcher to survey the frequency and prevalence of the off-label use of antineoplastic drugs and are also significant for hospital administrators, physicians, and patients. Our study proposed a new, more detailed classification and, for the first time, comprehensively expanded and provided illustrative examples of the off-label usage of antineoplastic drugs in the field of oncology. We argue that such a classification may encourage adoption in practice and improve the management of the use of antitumor off-label drug. Furthermore, we encourage research and constructive discussions with the goal of a new international consensus.
